# Removal of regulatory T cells prevents secondary chronic infection but increases the mortality of subsequent sub-acute infection in sepsis mice

**DOI:** 10.18632/oncotarget.7682

**Published:** 2016-02-24

**Authors:** Ruichao Huo, Lili Wang, Xiaoya Wang, Yong Zhao, Yi Wang, Xiaomin Zhao, Lingling Chang, Shan-lu Liu, Dewen Tong, Hai Zhang, Yong Huang

**Affiliations:** ^1^ College of Veterinary Medicine, Northwest A&F University, Yangling, Shaanxi, China; ^2^ Laboratory Animal Center, Fourth Military Medical University, Xi'an, Shaanxi, China; ^3^ Department of Molecular Microbiology and Immunology, Bond Life Sciences Center, University of Missouri, Columbia, MO, United States of America

**Keywords:** sepsis, immunosuppression, lymphocyte, immunotherapeutic approach, secondary infection, Immunology and Microbiology Section, Immune response, Immunity

## Abstract

The immunosuppression following initial septic insult impairs resistance to secondary infection. Modulation of lymphocytes population may help to develop an effective therapeutic strategy. In this study, lipopolysaccharide (LPS)-induced endotoxemia was employed as the initial septic insult. 24 hours later, mice underwent cecal ligation and puncture to induce chronic or sub-acute peritonitis. Potential usefulness of T regs deletion antibody (anti-CD25) in improving LPS-induced immunosuppression and the survival of subsequent different infections were evaluated. LPS injection induced lymphocyte loss and led to decreased IL-6, TNF-α and IFN-γ, and weakened bacteria clearance upon chronic peritonitis at 24 h post-LPS, whereas reconstitution with lymphocytes reversed these changes. LPS-induced T regs expansion contributed to T and NK cells decrease in number and activity during sepsis. Depletion of T regs using anti-CD25 antibodies partly prevented lymphocyte loss and increased the responses of T and NK cells to subsequent stimulation, resulting in significantly increased bacterial clearance and survival in a 2-hit model of chronic peritonitis, but which significantly increased early mortality upon subsequently sub-acute infection. Yet, using lower dosage of anti-CD25 antibodies to moderate down-regulate T regs levels could partly improve bacterial clearance and survival in either chronic or sub-acute infection. These results demonstrate that using anti-CD25 antibodies to deplete T regs can ameliorate immunosuppression through increasing T cells and NK cells responses in sepsis, which is beneficial for preventing subsequently chronic infection, but will probably bring some deleterious effects for subsequent sub-acute infection.

## INTRODUCTION

Sepsis, a disorder systemic inflammation response to severe infection or injury, is the leading cause of death in noncoromary intensive care units and the 11^th^ leading death cause in the United States [1, 2]. It is characterized by an initial hyperinflammatory phase termed the systemic inflammatory response syndrome (SIRS), followed by a hypoinflammatory or immunosuppressive phase termed the compensatory anti-inflammatory response syndrome (CARS) [2, 3]. Despite overall survival rate has been improved through using early goal-directed therapy in the past decades, sepsis continues as a major life-threatening disease in critical ill patients [4, 5]. It has been acknowledged that sepsis-related deaths are not restricted solely to the early phase SIRS, the late deaths remain frequent in critical ill patients and also observed in different animal models of sepsis [6, 7]. During the late phase of sepsis, patients or animal models are susceptible to secondary infections due to immune dysfunction, which will result in an increased microbiological burden and death risk [8, 9]. In recent years, late death occurred in immunosuppressive phase of sepsis often exceeds the death occurred in acute phase of sepsis [10]. Immunosuppression-associated immunopathologic alterations have been considered as a predominant factor in induction of late death in the majority of patients who survive the initial hypeinflammatory state, but immunopathologic alterations preceding immunosuppression are not yet well defined [7, 11]. Therefore, to determine the immunopathologic alterations preceding immunosuppression and to modulate inflammation response *via* selectively inhibiting the immunopathologic alterations have been widely considered as key steps and possible means for preventing life-threatening secondary hospital-acquired infection and improving survival, especially for late death of sepsis patients.

Regulatory T cells (T regs), a subset of CD4(+) CD25(+) T lymphocytes, play a pivotal role in maintenance of the balance of inflammatory response [12]. T regs have been shown to restrict aggressive immune responses, particularly as serve to prevent excessive CD4^+^ and CD8^+^ T cell responses. Several studies have shown that the percentages and numbers of circulating T regs are markedly increased in septic patients and animals, which presumably contributes to the development of immunosuppression in the later phase of sepsis [13-16]. Rat anti-mouse CD25 mAb (clone PC61) has been confirmed to cause sustained and selective depletion of T regs in mice lasting up to 7 days post injection, and widely applied in studying the roles of T regs in different fields [17-19]. However, the exact effects of depleting T regs by clone PC61 on sepsis outcome are not well defined.

In this study, we firstly determined the kinetic profiles of CD4^+^ and CD8^+^ T cells, NK cells, B cells and T regs over the time course of sepsis induced by lipopolysaccharide (LPS) injection and the effects of depleting T regs by clone PC61 on the numbers and activities of different lymphocytes in the development of immunosuppression. Furthermore, we used two kinds of two-hit models to assess whether down-regulation of T regs by clone PC61 could reverse immunosuppression, enhanced the resistance to subsequently different infections and improved the survival of septic mice. We aimed to identify the exact effects of clone PC61 on improving the immunosuppression and outcome of sepsis, and tried to explore an effective individualized treatment method for the certain type of sepsis patients through adjusting the levels of T regs.

## RESULTS

### LPS injection induces lymphocyte loss and leads to immunosuppression

Injection of 1.25 mg/kg LPS have been identified to induce sepsis with immunosuppression [20]. Mice injected with 5 mg/kg LPS (LPS mice) showed more serious clinical signs and extension of recovery times compared to the mice given 1.25 mg/kg LPS and did not resulted in death (Supplementary Figure 1).

**Figure 1:**
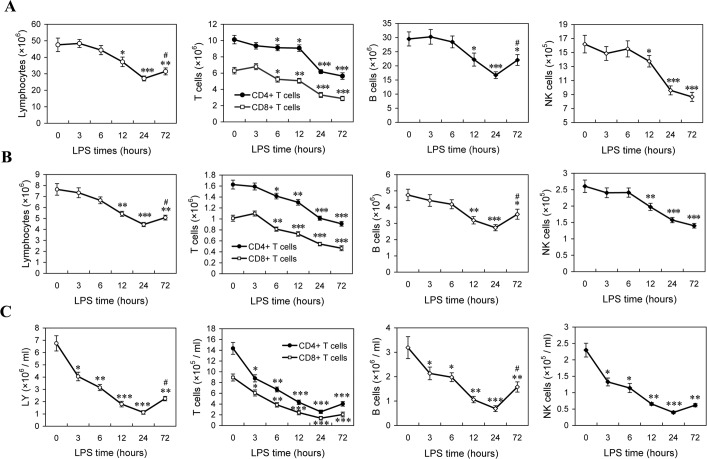
The changes of lymphocytes in LPS-induced sepsis mice Mice were injected with LPS (5mg/kg. *i.v*), and the absolute numbers of lymphocytes in the spleens **A.**, lymph node **B.** and peripheral blood **C.** at indicated times post-LPS were determined as described in Methods. The results are means ± SEM values of 3 independent experiments (*n* = 9). Data were analyzed by 1-way ANOVA with Bonferroni's multiple comparisons test; *p < 0.05, **p < 0.01, ***p < 0.001 *versus* the data at 0 h post-LPS. ^#^*p* < 0.05 *versus* the data at 24 h post-LPS.

In the mice injected with 5 mg/kg LPS, we detected the changes of lymphocytes in spleen, lymph node and peripheral blood. A detailed time-course showed that, after LPS challenge, the number of spleenic lymphocytes decreased at 12 h post-LPS and reached to the lowest point by 24 hours, followed by a reversed increase at 72 h post-LPS; CD4^+^ and CD8^+^ T cells reduced at 6 h post-LPS, and progressively declined at subsequent time points; B cells reduction was appeared at 12 h, and reached to the lowest point by 24 hours, followed by a reverse at 72 h post-LPS; NK cells were appeared to be a significant decrease at 12 h post-LPS and progressively declined at subsequent time points (Figure 1A). Similar changes were also found in the lymphocytes from inguinal lymph node and peripheral blood (Figure 1B and 1C). These results suggested that LPS injection induces lymphocyte loss, including CD4^+^ T cells, CD8^+^ T cells, B cells and NK cells, particularly in the late phase of sepsis.

**Figure 2:**
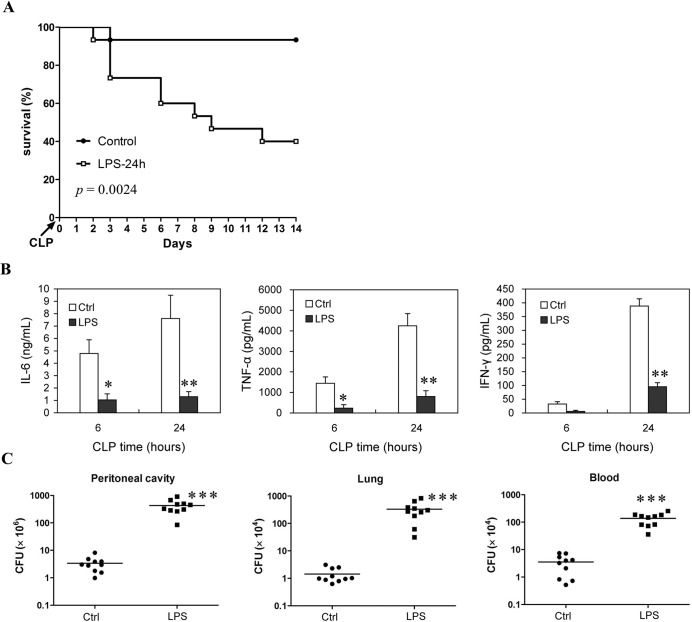
Survival rate and immune response ability of the LPS-induced sepsis mice when they are challenged with chronic CLP at the late phase of sepsis Mice injected with LPS (5mg/kg body weight, *i.v.*) or saline (Control) were challenged with chronic CLP at 24 h post-LPS (LPS-24h), then survival was observed for 14 days from CLP injury (10-15 mice/group, Kaplan-Meier plots followed by log-rank test) **A.**, the levels of IL-6, TNF-α and IFN-γ in serum were determined at 6 h and 24 h post-CLP **B.**, and tissue bacteria counts were measured 3 days after CLP **C.** Results are representative of three of independent experiments. Data in B and C were analyzed by 2-tailed Student's t test, n = 5-10 per group; *p < 0.05, **p < 0.01, ***p < 0.001 *versus* Ctrl group at same time **B.** or in same tissue **C.**

To determine the ability to develop immune response of the LPS-injected mice in the late phase of sepsis, a chronic peritonitis induced by CLP using a 30-gauge needle was used as second-hit, which showed an over 90% survival at 14 days post-CLP in the control mice, whereas mice receiving this chronic CLP at 24 h post-LPS showed a 40% survival at 14 days post-CLP (Figure 2A). In addition, compared with the control mice, LPS mice also exhibited a decreased induction of IL-6, TNF-α and IFN-γ (Figure 2B) and weakened bacteria clearance ability upon challenging with the chronic CLP at 24 h post-LPS (Figure 2C). The bacterial counts in the peritoneal cavity, blood and lungs increased about two orders of magnitude in the LPS-injected mice challenged with the chronic CLP procedure compared to that in control mice challenged with chronic CLP procedure (Figure 2C). Likewise, mice given 5 mg/kg of LPS followed by challenging with non-lethal doses of *Escherichia coli* or *Staphylococcus aureus* at 24 h post-LPS exhibited reduced bacteria clearance compared with the control mice challenged with same bacteria infection (Supplementary Figure 2). Taken together, these data demonstrated that injection of 5 mg/kg LPS induces lymphocyte loss in mice, which may be associated with the relatively lower ability in cytokines production and bacteria clearance upon secondary infection.

### Reconstitution of lymphocytes enhances resistance to subsequent infection and improves survival of sepsis mice

**Figure 3:**
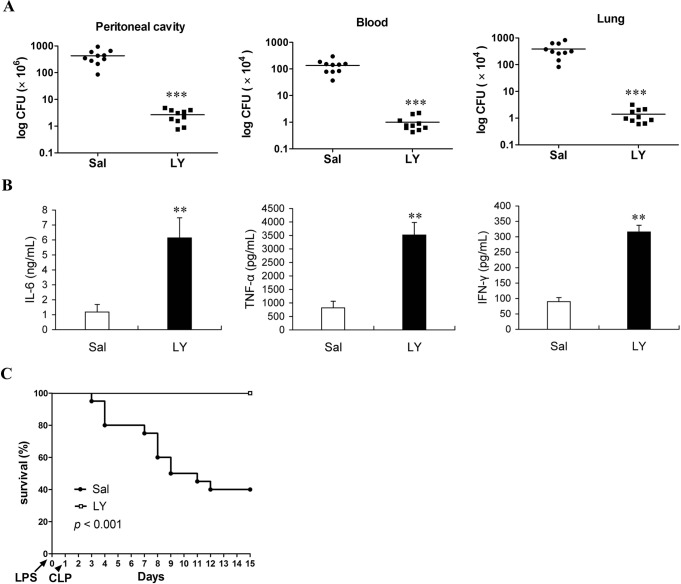
Infusion of lymphocytes can effectively enhance resistance to subsequent infection and improve survival of sepsis mice undergoing second-hit Mice were challenged with chronic CLP at 24 h post-LPS, followed by infusion of lymphocytes (LY) or saline (Sal) at 6 hours post-CLP, then tissue bacteria counts were measured 3 days post-CLP **A.**, the levels of IL-6, TNF-α and IFN-γ in serum were determined at 24 h post-CLP **B.**, survival was observed for 14 days from CLP and analyzed by Kaplan-Meier plots with log-rank test **C.** Results are representative of two of independent experiments. Data were analyzed by 2-tailed Student's t test, n = 5-10 per group (for A, B); **p < 0.01, ***p < 0.001 *versus* the group without treatment (Sal).

To confirm whether the loss of lymphocytes play a pivotal role in promoting the development of immunosuppression in sepsis, lymphocytes isolated from same litters of mice were infused into the peripheral blood of the LPS mice undergoing CLP (Note that the number of infused lymphocytes was about 1×10^7^). As predicted, infusion of lymphocytes into LPS mice at 6 h post-CLP could partly replenish the lost lymphocytes (Supplemental Figure 3), and significantly decreased bacterial counts in the peritoneal cavity, blood and lungs by greater than two orders of magnitude compared to the same tissues from the same first- and second-hit mice that were infused with same volume of 0.9% saline (Figure 3A). Consistent with the enhanced resistance to secondary infection, after challenging with chronic CLP, the serum cytokines levels were also significantly increased in the LPS mice infused with lymphocytes compared with that in the LPS mice infused with same volume of 0.9% saline (Figure 3B). Consequently, infusion of lymphocytes increased over 50% of survival in the LPS mice undergoing chronic CLP (Figure 3C). These results suggested that reconstitution of lymphocytes population improves the ability of immune response in the immunosuppressive phase of sepsis mice and enhances host resistance to subsequent infections.

### LPS injection-induced T regs expansion leads to decreases of T cells and NK cells in the late phase of sepsis

**Figure 4:**
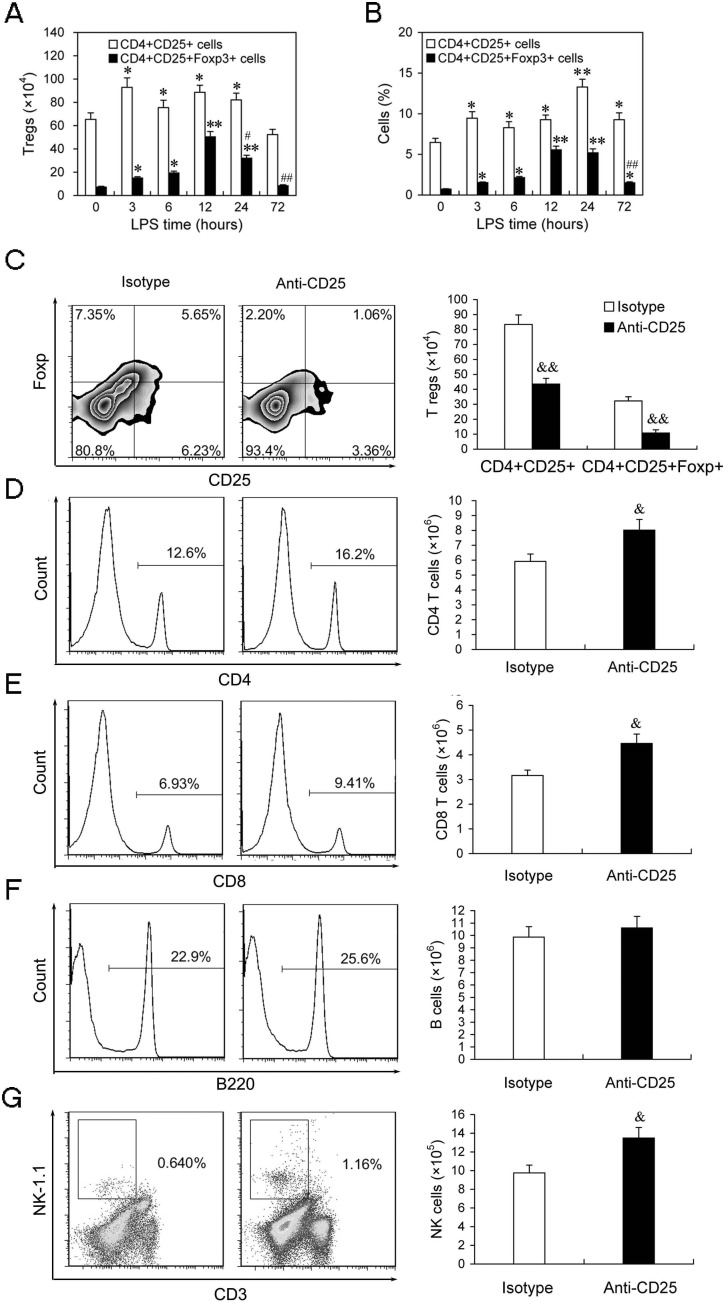
The expansion of T regs is involved in regulation of T cells and NK cells number in LPS-induced sepsis mice **A.**,**B.** The absolute number and percentage of T regs in LPS-injected mice. Mice were injected with LPS (5 mg/kg body weight, *i.v*.), and the absolute numbers and percentages of CD4^+^CD25^+^ and CD4^+^CD25^+^Foxp3^+^ cells in the spleens at indicated times post-LPS were determined by flow cytometry. The results are means ± SEM values of 3 independent experiments (n = 6). Data were analyzed by 1-way ANOVA with Bonferroni's multiple comparisons test; *p < 0.05, **p < 0.01 *versus* the same cells population at 0 h post-LPS. ^#^*p* < 0.05, ^##^*p* < 0.01 *versus* the same cells population at 12 h post-LPS. **C.**-**G.** Depletion of T regs partly increased the percentages of T and NK cells in the late phase of sepsis. Group of mice were injected *i.p.* with anti-CD25 mAb or isotype-matched control mAb (100 μg/mouse) at the same time of LPS challenge. After 24 h, animals were sacrificed and splenocytes were assessed for the percentages and absolute numbers of CD4^+^CD25^+^ and CD4^+^CD25^+^Foxp3^+^ cells **C.**, CD4^+^ T cells **D.**, CD8^+^ T cells **E.**, B220^+^ B cells **F.** and CD3^−^NK-1.1^+^ NK cells **G.** in spleen. Results are representative of two independent experiments. Data were analyzed by 2-tailed Student's t test and shown as mean ± SD, 4-6 mice/group; ^&^*p* < 0.05, ^&&^*p* < 0.01 *versus* isotype Abs treatment group.

To investigate the roles of T regs in the development of sepsis, the absolute numbers and percentage of CD4^+^CD25^+^ and CD4^+^CD25^+^Foxp3^+^ T cells were measured firstly at different time points in LPS-injected mice. The absolute numbers of CD4^+^CD25^+^ T cells increased from 3 h to 24 h, while CD4^+^CD25^+^Foxp3^+^ T cells started increasing at 3 h, peaked by 12 h, followed by a decline at 24 h and 72 h post-LPS (Figure 4A), while the percentage of CD4^+^CD25^+^ T cells and CD4^+^CD25^+^Foxp3^+^ T cells in CD4^+^ T cells showed similar change patterns as their absolute numbers after injection of LPS (Figure 4B), suggesting that LPS injection induced T regs expansion.

To better understand the impact of T regs expansion on the changes of lymphocytes in the development of immunosuppression in the late phase of sepsis, the changes of lymphocytes were determined in the mice that T regs were depleted by anti-CD25 antibodies. Since T regs-deficient mice are susceptible to lower doses of LPS that do not enough to induce sepsis [17, 21], thus T regs were depleted by injection of anti-CD25 mAb in LPS-challenged mice in this study. As expected, depletion of T regs by injection of anti-CD25 mAb (100 μg/mouse) rendered CD4^+^CD25^+^Foxp3^+^ T cells to decrease greater than 80% at 24 h post-injection LPS (Figure 4C). The percentages and numbers of CD4^+^ T cells, CD8^+^ T cells and NK cells were significantly increased in the splenocytes of mice injected with anti-CD25 mAb compared with those in isotype control-treated mice (Figure 4D, 4E & 4G), whereas B cells increased partly but did not show significant difference (Figure 4F).

### Depletion of T regs increases the activities of T cells and NK cells in the late phase of sepsis

**Figure 5:**
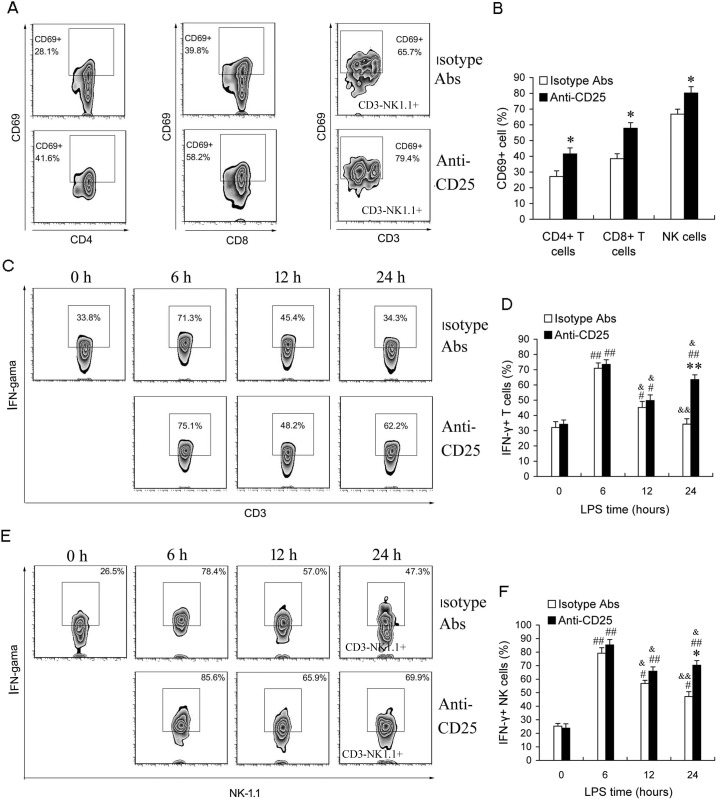
Depletion of T regs increases the activation of T and NK cells at the late phase of sepsis in LPS-induced sepsis mice Group of mice were injected *i.p.* with anti-CD25 mAb or isotype-matched control mAb (100 μg/mouse) at the same time of LPS challenge. **A.**, **B.** After 24 h, animals were sacrificed, and splenocytes were assessed for CD4^+^CD69^+^, CD8^+^CD69^+^ activated T cells and CD3^−^NK1.1^+^ CD69^+^ activated NK cells. **C.**-**F.** Animals were sacrificed at 0 h, 6 h, 12 h, and 24 h post-LPS, and whole splenocytes were assessed IFN-γ production directly *ex vivo* following 6 h *in vitro* stimulation with IL-12 (1 ng/ml). Results are representative of three of independent experiments. Data are shown as mean ± SD (SEM), 4-6 mice/group, 2-tailed Student's t test or 1-way ANOVA with Bonferroni's multiple comparisons test; *p < 0.05, **p < 0.01 *versus* isotype Abs treatment group at same time or in same cells. ^#^*p* < 0.05, ^##^*p* < 0.01 *versus* the data of same group at 0 h post-LPS. ^&^*p* < 0.05, ^&&^*p* < 0.01 *versus* the data of same group at 6 h post-LPS.

To further identify the impact of T regs expansion on the activities of lymphocytes in the late phase of sepsis, we compared the percentage of CD69^+^ cells and IFN-γ-producing cells in T cells and NK cells in either T regs-depleted LPS mice or isotype control Abs-treated LPS mice. Splenocytes from T reg-depleted sepsis mice (given 100 μg anti-CD25 mAb) contained higher percentages of CD4^+^CD69^+^, CD8^+^CD69^+^ activated T cells and CD3^−^NK1.1^+^ CD69^+^ activated NK cells compared with isotype matched controls (Figure 5A & 5B). Upon IL-12 stimulation *in vitro*, the percentages of IFN-γ-producing T cells and NK cells significantly increased at 6 h post-LPS, then declined at 12 h post-LPS in either anti-CD25 mAb-treated sepsis mice or isotype control mAb-treated sepsis mice, and further decreased in isotype control mAb-treated sepsis mice at 24 h post-LPS, but did not further decrease in anti-CD25 mAb-treated sepsis mice at 24 h post-LPS (Figure 5C-5F). Noteworthily, the percentage of IFN-γ-producing T cells and NK cells in anti-CD25 mAb-treated sepsis mice were higher than that in isotype control mAb-treated sepsis mice at 24 h post-LPS (Figure 5C-5F). These results suggested that LPS-induced T regs expansion inhibits the activation and response of T cells and NK cells in the late phase of sepsis, and anti-CD25 mAb-treatment can efficiently improve the immune status of the late phase of sepsis.

### Massive depletion of T regs enhances resistance to subsequent chronic infection and improves survival rate but increases early mortality of subsequent sub-acute infection

**Figure 6:**
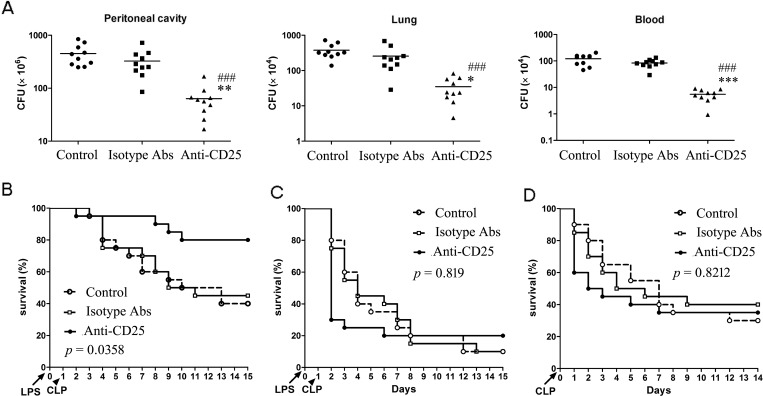
Depletion of T regs significantly enhances bacteria clearance and improves total survival rate of LPS-induced sepsis mice when challenged with chronic peritonitis, but significantly increases early mortality when challenged with sub-acute peritonitis **A.**, **B.** Group of mice were injected *i.p.* with anti-CD25 mAb or isotype-matched control mAb (100 μg/mouse) at the same time of LPS challenge, or without Abs treatment (Control), followed by a CLP to induce chronic peritonitis at 24 h post-LPS. Tissue bacteria counts were measured 3 days after chronic CLP **A.**, and survival was observed for 15 days from LPS challenge **B. C.** The LPS mice were treated with anti-CD25 mAb or isotype-matched control mAb as A, followed by a CLP to induce sub-acute peritonitis. Survival was observed for 15 days from LPS challenge. **D.** The normal mice were injected *i.p*. with anti-CD25 mAb or isotype-matched control mAb (100μg/mouse), or without Abs treatment (Control), and followed by a CLP at 24 h after mAb injection to induce sub-acute peritonitis. Survival was observed for 14 days from CLP (10-15 mice/group, Kaplan-Meier plots followed by log-rank test). Results are representative of three of independent experiments. Data in A were analyzed by 1-way ANOVA with Bonferroni's multiple comparisons test, n = 10 per group. *p < 0.05, **p < 0.01, ***p < 0.001 versus isotype Abs treatment group; ^###^*p* < 0.001 *versus* Control group without Abs treatment.

Given massive depletion of T regs could increase the activation and response of T cells and NK cells in the late phase of sepsis, we next identified whether massive depletion of T regs could enhance resistance to subsequent infections. Firstly, we detected the bacterial clearance rate of T regs-depleted LPS mice undergoing a chronic peritonitis induced by CLP (30-gauge needle, one puncture) at 24 hours post-LPS. Treatment with 100 μg/mouse anti-CD25 mAb at the same time of LPS challenge decreased bacterial counts in the peritoneal cavity, blood and lungs after second-hit about one order of magnitude compared to the same tissues from LPS mice that received isotype control Abs treatment or control LPS mice without treatment (Figure 6A). Consequently, chronic peritonitis induced by CLP resulted in approximate 60% mortality at 14 days post-CLP in LPS mice treated with isotype control Abs, whereas T regs-depletion increased the survival of LPS mice over 30% (*p* = 0.0358) (Figure 6B). These results suggested that depletion of T regs enhances resistance to subsequent chronic infections and improves the survival rate of LPS sepsis mice upon second chronic infections.

Next, we asked that whether massive depletion of T regs was always beneficial for the sepsis mice resistance to second-hit. We then evaluated the survival of LPS mice given 100 μg/mouse anti-CD25 mAb or isotype control mAb after undergoing a medium-grade CLP severity (25-gauge needle, double puncture). This medium-grade CLP challenge could induce a sub-acute peritonitis and resulted in an about 20%, 60% and 90% mortality in the LPS mice by day 1, day 4, and day 14 post-CLP, respectively (Figure 6C). However, given 100 μg of anti-CD25 mAb doubled the mortality of LPS mice by day 1 after undergoing the sub-acute CLP (*p* < 0.01), and did not significantly decrease the overall mortality at 14 days post-CLP when compared with that in the LPS mice treated with isotype control Abs or control LPS mice without treatment (*p* = 0.819) (Figure 6C). Consistent with this change, T regs-depleted mice also appeared an increased early mortality (*p* < 0.01) and an unchanged overall survival rate (*p* = 0.8212) when they underwent sub-acute CLP alone (Figure 6D). These results suggested that massive depletion of T regs might lead to potentially fatal hyperinflammatory upon subsequently severe infection.

### Moderate down-regulation of T regs level benefits to control the mortality of subsequent sub-acute infection and improves total survival of sepsis mice

**Figure 7:**
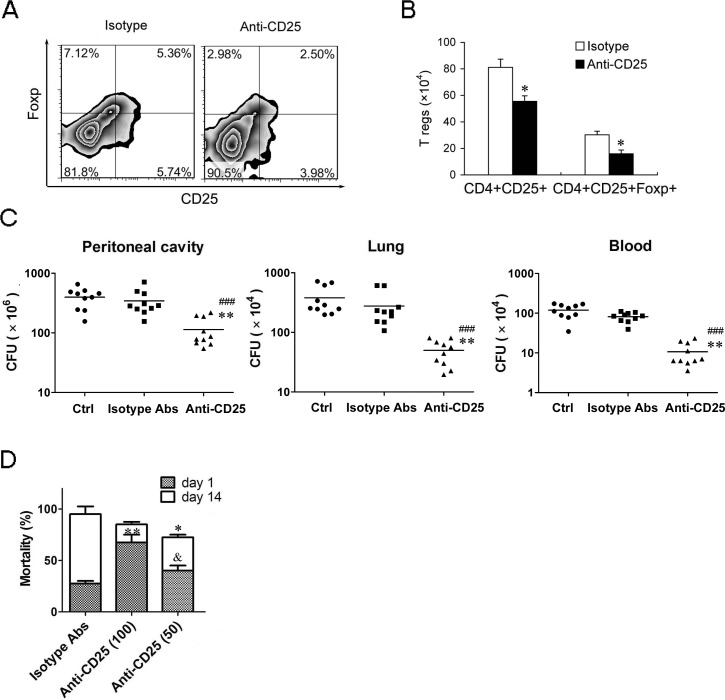
Moderate down-regulation of T regs level can effectively enhance the bacteria clearance and decreases the mortality of subsequent sub-acute infection **A.**, **B.** The percentage and absolute number of T regs in LPS mice injected with anti-CD25 mAb or isotype-matched control mAb (50 μg/mouse). **C.** Group of mice were injected *i.p.* with anti-CD25 mAb or isotype control Abs (50 μg/mouse) at the same time of LPS challenge, or without Abs treatment (Ctrl), followed by a chronic CLP challenge at 24 h post-LPS. Tissue bacteria counts were measured 3 days after chronic CLP. **D.** Group of mice were injected *i.p.* with anti-CD25 mAb (50 or 100 μg/mouse) or isotype control Abs at the same time of LPS challenge, followed by a sub-acute CLP challenge at 24 h post-LPS, and mortality was observed for 14 days from CLP challenge. Results are representative of three of independent experiments. Data were analyzed by Student's t test (for B) or 1-way ANOVA (for C & D), n = 10 per group. *p < 0.05, **p < 0.01 *versus* isotype Abs treatment group in same cells **B.**, same tissue **C.** or on same days **D.**; ^###^*p* < 0.001 *versus* Control group without Abs treatment (Ctrl); ^&^*p* < 0.05 *versus* Anti-CD25 Abs (100 μg/mouse) treatment group at day 1 post-CLP.

To identify whether moderate down-regulation of T regs levels could enhance resistance to subsequent chronic infections and reduce susceptibility to secondary severe infection. We used 50 μg/mouse of anti-CD25 mAb to induce about 50% T regs depletion (Figure 7A & 7B). After undergoing chronic CLP, bacterial counts in the peritoneal cavity, blood and lungs decreased 2.02-folds, 4.54-fold and 6.62-fold, respectively, in the LPS mice injected 50 μg anti-CD25 mAb compared to those in the LPS mice received isotype control Abs treatment (Figure 7C). After undergoing a sub-acute CLP, the mortality of day 1 post-CLP was significantly decreased in the LPS mice given 50 μg anti-CD25 mAb compared to that in the LPS mice given 100 μg anti-CD25 mAb, while overall mortality of 14 days post-CLP in the LPS mice given 50 μg anti-CD25 mAb was also lower than that in the LPS mice given isotype control Abs (Figure 7D). These data suggest that moderate down-regulation of T regs benefits to control the mortality of subsequent sub-acute infection and improve total survival of sepsis mice.

## DISCUSSION

Patients who survive severe SIRS display compromised immune response that contributes to the lethality of subsequent infection or trauma at the late phase of sepsis [10, 11]. Previous works in animal models as well as patients who suffered severe sepsis have shown that sepsis induces extensive lymphocyte loss *via* apoptosis [15, 16, 22]. The degree of sepsis-induced lymphocyte loss observed in human correlated with disease severity attributing to sepsis-assoicated immunoparalysis [16, 23]. In this study, we found that a certain dose of LPS also could induce an immunosuppressive phenotype consisting of systemic lymphocyte loss and decreased responsiveness of lymphocytes, which was consistent with the changes occurred in those trauma patients who developed infection or death due to a significantly decreased circulating lymphocytes [24]. The data in present study also demonstrated that transfer of normal murine lymphocyte into LPS-induced sepsis mice to replenish the lost lymphocytes can improve immune function and enhance resistance to secondary chronic infection. These results provided direct evidences for the correlation of lymphocyte loss with sepsis-assoicated immunoparalysis, and suggested that modulation of lymphocytes may be effective method for the treatment of sepsis.

It is well known that lymphocytes are activated to produce proinflammatory cytokines and activate macrophages to combat pathogens in the early stage of sepsis. As sepsis processes, sepsis induces apoptosis of critical immune cells of immune system leading to immunologic response shift to a hypoinflammatory response [15]. Despite loss of lymphocytes in sepsis may be detrimental by impairing the ability of the immune system to combat pathogens, loss of lymphocytes may be beneficial to preventing potentially fatal hyperinflammatory by down-regulating the excessive inflammatory response in the early phase of sepsis, particularly in the onset phase of severe sepsis [15]. Therefore, lymphocytes loss may be an important mechanism in tuning the balance of inflammatory response. Meanwhile, T regs have been shown to restrict aggressive immune response and play a major role in maintenance of peripheral tolerance, and defects in T reg number and function have been linked to hypersensitivity upon infection and result in mortality to an otherwise nonlethal infection [17]. Previous studies have shown that regulatory T cells play important roles in the progression of developing immune dysfunction in experimental sepsis [25, 26]. In this study, our results also highlighted the contribution of T regs expansion to the development of immunosuppression, and identified the roles of T regs in regulating the numbers and activities of CD4^+^, CD8^+^ T cells and NK cells in sepsis. In our two-hit models, massive removal of T regs could block the development of immunosuppressive phenotype by partly inhibiting lymphocytes loss and reversing the reduction of T cell and NK cell activities and immune response in the late phase of sepsis. These results not only further support the concept that lymphocyte loss is a major reason for the development of immunosuppressive phenotype in sepsis, but also demonstrate that T regs expansion is an important mechanism for the development of immunosuppressive phenotype in sepsis.

In this study, we also found that despite using lager dosage of anti-CD25 mAb to deplete CD4^+^CD25^+^ T regs enhanced resistance to subsequent infections and improved the survival of sepsis mice when challenged with secondary chronic peritonitis, this approach significantly increased the early death in sepsis mice when challenged with sub-acute peritonitis due to potentially fatal hyperinflammatory to subsequent severe infection. Recently, another study shows that DEREG mice depleted of Foxp3^+^ T reg cells exhibit higher disease scores and mortality rates than non-depleted DEREG mice in the early-phase of severe sepsis, which similarly indicates that Foxp3^+^ T reg cells limit the hyper-inflammatory response [27]. Anyhow, removal of T regs using different methods may represent a novel strategy for improving the immunosuppression in the late phase of sepsis [26, 28]. However, we need notify that T regs expansion is also an important mechanism for preventing potentially fatal hyperinflammatory, and that the therapeutic approaches targeting to T regs also need pay more attention to the time of treatment and the kinds of secondary infections. The failure of anti-sepsis treatments in the past is most likely related to wrong timing of the treatment due to missing either reliable biomarkers to assess the condition of the patients or lack of awareness to potential secondary infection [29, 30]. In addition, because CD25 expression is also upregulated in non-regulatory CD4^+^ T cells after activation, CD25 antibodies also affect these cells. Thus, depletion of T regs using CD25 antibodies may bring some unpredictable dangers and some uncertain in the therapeutic outcome due to the subsequently unpredictable infection and the variant targets of CD25 antibodies. Collectively, although previous studies and our results suggested that using anti-CD25 Abs to deplete T regs can improve immune status of sepsis [17, 19, 31], we need further explore specific antibodies or drugs to deplete Foxp3^+^ Treg cells, and need exactly determine the time of treatment and the level of T regs according to the condition of sepsis patients when using this therapeutic approach to treat sepsis in future.

In summary, the present data in this study provide certain evidences for the advantages and disadvantages of using anti-CD25 Abs to remove T regs in improving the immunosuppression of sepsis and preventing variant secondary infection. Further deeply understanding of the regulation mechanisms of T regs in sepsis may help to design more effective immune-based therapies to improve the outcome of post septic patients.

## MATERIALS AND METHODS

### Antibodies and reagents

Abs against following antigens: CD3-FITC, CD3-APC, CD25-FITC, CD4-PE, CD4-PECy7, CD8-PE, B220-PE, NK-1.1-PE, CD69-APC, FOXP3-PE, IFN-γ-APC, Isotype-matched control Ab, Annexin V-FITC, 7AAD, Purified anti-CD25 mAb (PC61) were purchased from BioLegend (San Diego, CA). The murine recombinant cytokines (IL-2, IL-12) were from Peprotech (Rocky Hill, NJ).

### Animal

Six- to Eight-wk-old male C57BL/6 mice (Lab animal center, the forth military medical University) with an average weight of 20 g were used in this study. All mice were maintained in a specific-pathogen free environment at the Central Animal Care Services. All experiments were performed in accordance with the protocols approved by the Forth Military Medical University Instituional Animal Care and Use Committee (IACUC).

### *In vivo* LPS-induced Sepsis model

LPS-induced sepsis model was employed as the initial septic insult. Male C57BL/6 mice, 17 to 23 g body weight and 6 to 8 weeks of age, were housed for at least 1 week prior to use. To induce nonlethal and lethal systemic inflammation, mice were injected *i.v.* with LPS (1.25 - 20 mg/kg of body weigh; *Escherichia coli* serotype 055.B5; Sigma-Aldrich) and were monitored for 7 days [20]. Mice injected with LPS were monitored periodically for clinical signs and were blindly scored to indicate disease severity. Mice were monitored for movement, alertness, body temperature, body weight and others. Disease severity was scored in a semiquantitative fashion as previously described [21]. As follows: 0, no abnormal clinical sign; 1, ruffled fur but lively; 2, ruffled fur, moving slowly, hunched, and sick; 3, ruffled fur, squeezed eye, hardly moving, down, and very sick; 4, moribund; and 5, dead. A clinical score of 4 was used as the humane endpoint because the institutional ethical regulation does not permit score 5 in all animal experiments.

### The “second-hit”

a) The “second-hit” chronic CLP model of sepsis

The CLP model as developed by Baker et al. and Muenzer et al. was used to induce chronic intra-abdominal peritonitis as described previously [32, 33]. After injected *i.v.* with 5 mg/kg LPS or vehicle for 24 h, the mice were anesthetized with isoflurane (5% induction, 2% maintenance), and a midline laparotomy was performed. The distal one-third of the cecum was ligated and punctured once with a 30-gauge needle. This level of injury was utilized to create a prolonged infection with relatively low mortality (< 10%). The abdomen was closed in two layers, and 1 ml of 0.9% saline mixed with 0.05 mg/kg of body weight buprenorphine (Hospira Inc., Lake Forest, IL) was administered subcutaneously (s.c.) to replace third-space losses and provide pain control. A single dose of imipenem (25 mg/kg) was given s.c. 1 h post-CLP. One day post-CLP, 1 ml of 0.9% saline was given s.c. to all animals.

b) The “second-hit” sub-acute CLP model of sepsis

Following LPS challenge-induced initial septic insult, a medium-grade CLP severity (25-gauge needle, double puncture) was used to create a sub-acute infectious peritonitis with about 30% - 40% mortality during early sepsis. Except the needle was different in this model, the CLP protocol and treatment were followed previous studies [34, 35], and all animals were followed for 14 days or until death, whichever occurred first. Sham surgeries were not performed because we were comparing the response of LPS mice to the CLP.

c) The “second-hit” bacteria infection

At 24 h post-LPS, the mice were injected *i.v.* with non-lethal dose of *Escherichia coli* (5.8×10^6^ CFU) or *Staphylococcus aureus* (5.6×10^6^ CFU) to induce bacteremia. Mice were killed at 24 hours after bacteria infection, and then counted the bacterial clone number in the spleens, lungs and liver tissue samples to assess bacteria burden.

### Bacterial clearance

The mice were killed 3 days after chronic CLP, lungs tissue samples were aseptically excised, weighed, and homogenized in sterile saline using sterile tissue grinders. Blood samples were obtained by direct cardiac puncture using sterile technique. Peritoneal fluid were obtained by injection of 1 ml of sterile saline into peritoneal cavity and gently aspirated. Serial dilution of tissue homogenates, blood and peritoneal lavage fluid were plated on nutrient agar and incubated overnight at 37°C. Bacterial colony was counted to assess bacterial burden.

### T regs *in vivo* depletion

To deplete T regs *in vivo*, mice were injected with 100 μg or 50 μg Rat anti-mouse CD25 mAb (clone PC61) or isotype-matched control Ab (rat IgG1) *i.p.* at the indicated times after LPS challenge. 100 μg Rat anti-mouse CD25 mAb has been previously shown to cause sustained and selective depletion of T regs in mice lasting up to 7 d post-injection [17-19]. The degree of depletion was assessed by measuring spleen CD4^+^CD25^+^Foxp3^+^ cells by flow cytometry.

### Isolation of lymphocyte

After excision, spleens were placed in ice-cold PBS and passed through nylon mesh (200 μ m, Fisher), and washed with PBS contained 0.5% BSA and pelleted (300 g for 10 minutes at 4°C) to isolate splenocytes. Erythrocytes were lysed by erythrocyte lysis kit (R&D Systems). The remaining splenocyte pellet was resuspended in isolation buffer and further isolated lymphocytes by Mouse percoll (Sigma) following with the manufacturer's instructions. Fresh isolated lymphocytes from mice spleen were resuspended in PBS contained 0.5% BSA at a concentration of 1× 10^7^ cells/ml, followed by cell staining or *in vitro* assay.

### Quantification of absolute cell counts

Total cell counts per spleen or lymph node, or per ml blood were determined *via* the Vi-cell counter (Beckman Coulter). The percentages of CD4^+^ T, CD8^+^ T cells, B cells, NK cells and T regs etc., were determined *via* flow cytometric analysis (BD FACScan) as described previously [16]. The absolute cell counts for each splenic subset population were calculated by the following formula: cell counts of cell subpopulations = total cell counts multiplied by the subset population percentage.

### Flow cytometry and cytokines analysis

Cell surface Ags were labeled in staining buffer (Biolegend) with Abs at the concentration suggested by the manufacturers. Intracellular staining of IFN-γ, Foxp3 was performed after fixation and permeabilization buffer (Biolegend) treatment according to the manufacturer's instructions. All flow cytometry data were acquired and analyzed on flowJo v7.6 sofware as described previously [36, 37]. Cytometric Bead Array (CBA) Mouse Inflammation Kit (BD Bioscience, San Jose, CA) were used to detected plasma TNF-α, IL-6 and IFN-γ by flow cytometry.

### Statistics

All experiments were performed at least three times using at least *n* = 3 mice per assay, and results are representative of three of independent experiments. Prism software (GraphPad Software) was used for statistical evaluation. Data were expressed as means ± SEM (SD). Data were analyzed by ANOVA and Bonferroni post-hoc test, while comparisons between 2 groups were performed by unpaired Student's t test. Survival curves were analyzed by log-rank test. Statistical significance was defined as *P* < 0.05.

## SUPPLEMENTARY MATERIAL FIGURES


