# Supplementary material for: Removal of regulatory T cells prevents secondary chronic infection but increases the mortality of subsequent sub-acute infection in sepsis mice
Source: Oncotarget. 2016 Feb 24;7(10):10962–75. doi: 10.18632/oncotarget.7682 (PMC4905451; doi:10.18632/oncotarget.7682)
Supplement: Supplementary file 1 [file oncotarget-07-10962-s001.pdf]

Removal of regulatory T cells prevents secondary chronic infection but increases the mortality of subsequent sub-acute infection in sepsis mice

Supplementary Material

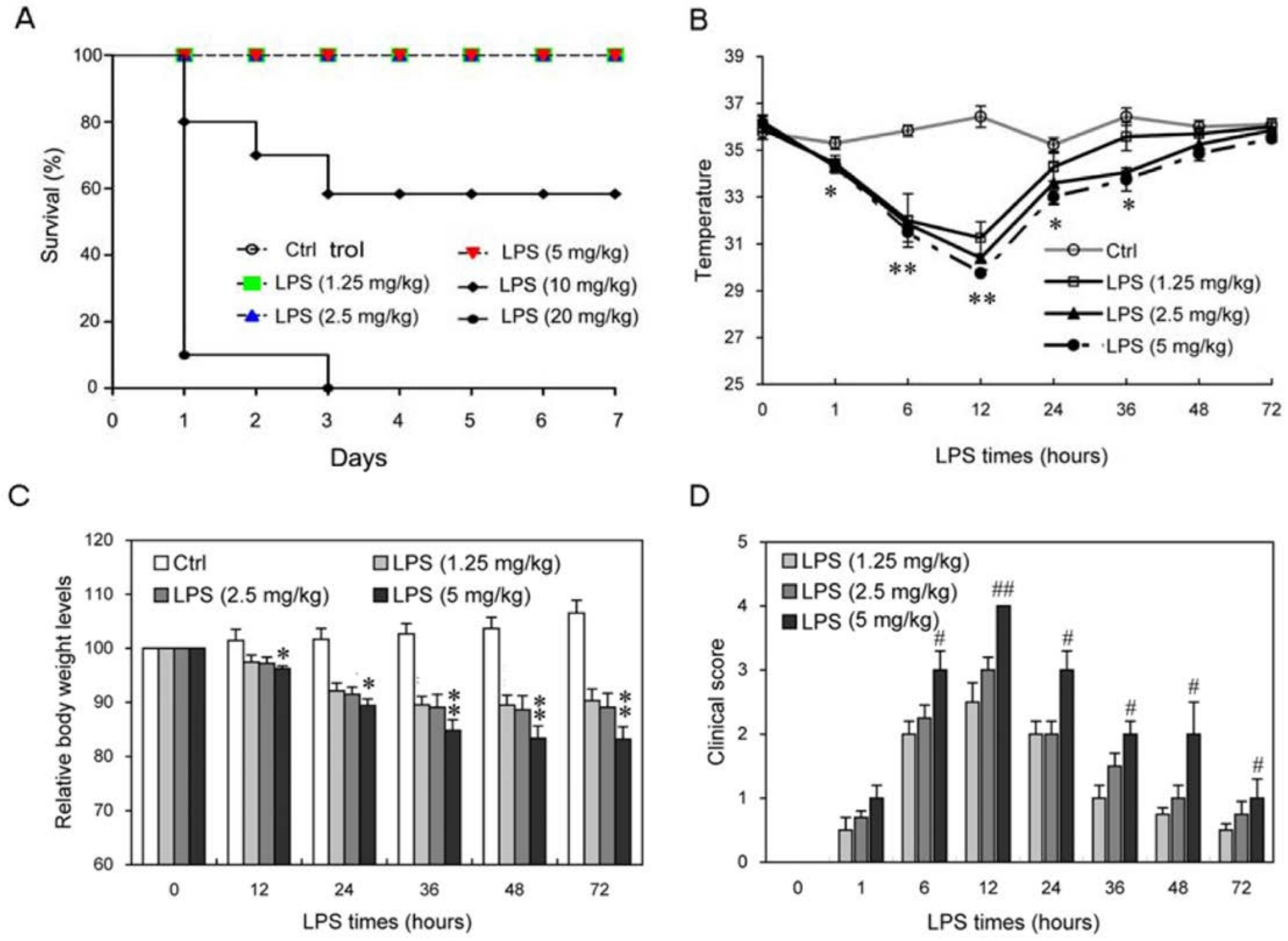

Supplementary Figure 1: The clinical signs and survival of the mice injected with indicated doses of LPS. (A) Mice injected with1.25, 2.5 or 5 mg/kg body weight of LPS did not result in lethal, whereas mice given 10 and 20 mg/kg LPS showed certain extent of mortality (8-10 mice/group, Kaplan-Meier plots followed by log-rank test). (B) Mice injected with 5 mg/kg of LPS appeared a lower body temperature and slow temperature recovery. (C) Injection of 5 mg/kg LPS caused a more significant body weight reduction. (D) Injection of 5 mg/kg LPS caused more serious clinical sign and extension of recovery times. Data were analyzed by 1-way ANOVA with Bonferroni's multiple comparisons test, n=3 per group; $^{*}p < 0.05$, $^{**}p < 0.01$ versus the Ctrl group at same time, $^{\#}p < 0.05$, $^{\#\#}p < 0.01$ versus the LPS (1.25 mg/kg) group at same time.

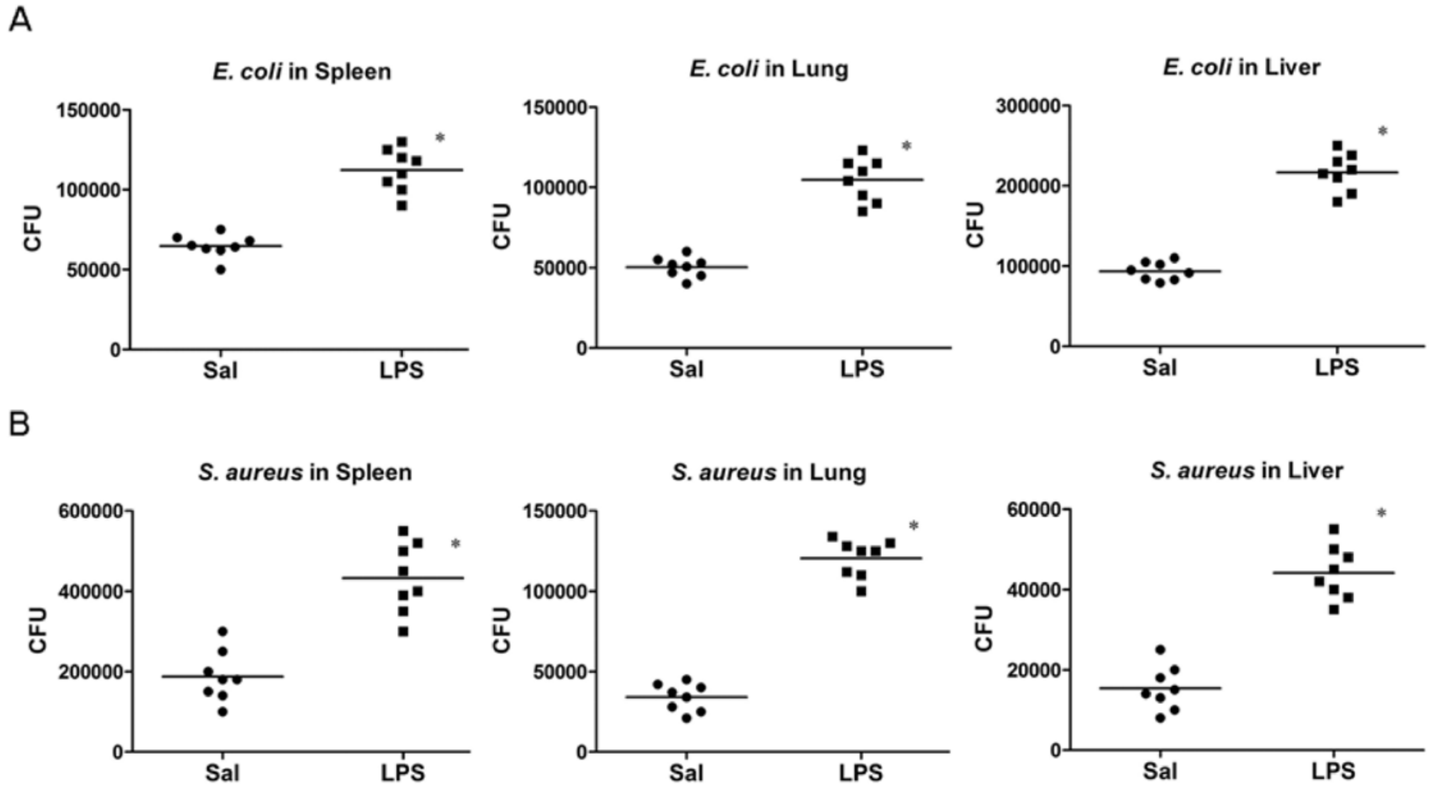

Supplementary Figure 2: Bacteria clearance of LPS-induced sepsis mice after *E. coli* or *S.aureus* challenge. Mice were injected with LPS (5mg/kg. i.v) and challenged with *E. coli* (A) or *S. aureus* (B) via *i.v.* at 24 h post-LPS. Tissue bacteria counts were measured at 24 h after bacteria challenge. Bacteria burden were significantly increased in the mice injected with LPS followed by bacteria infection compared with the saline-injected control group (Sal) followed by bacteria infection. Data are analyzed by 2-tailed Student's t test, n=8 per group; $^{*}p < 0.05$ versus the Sal group.

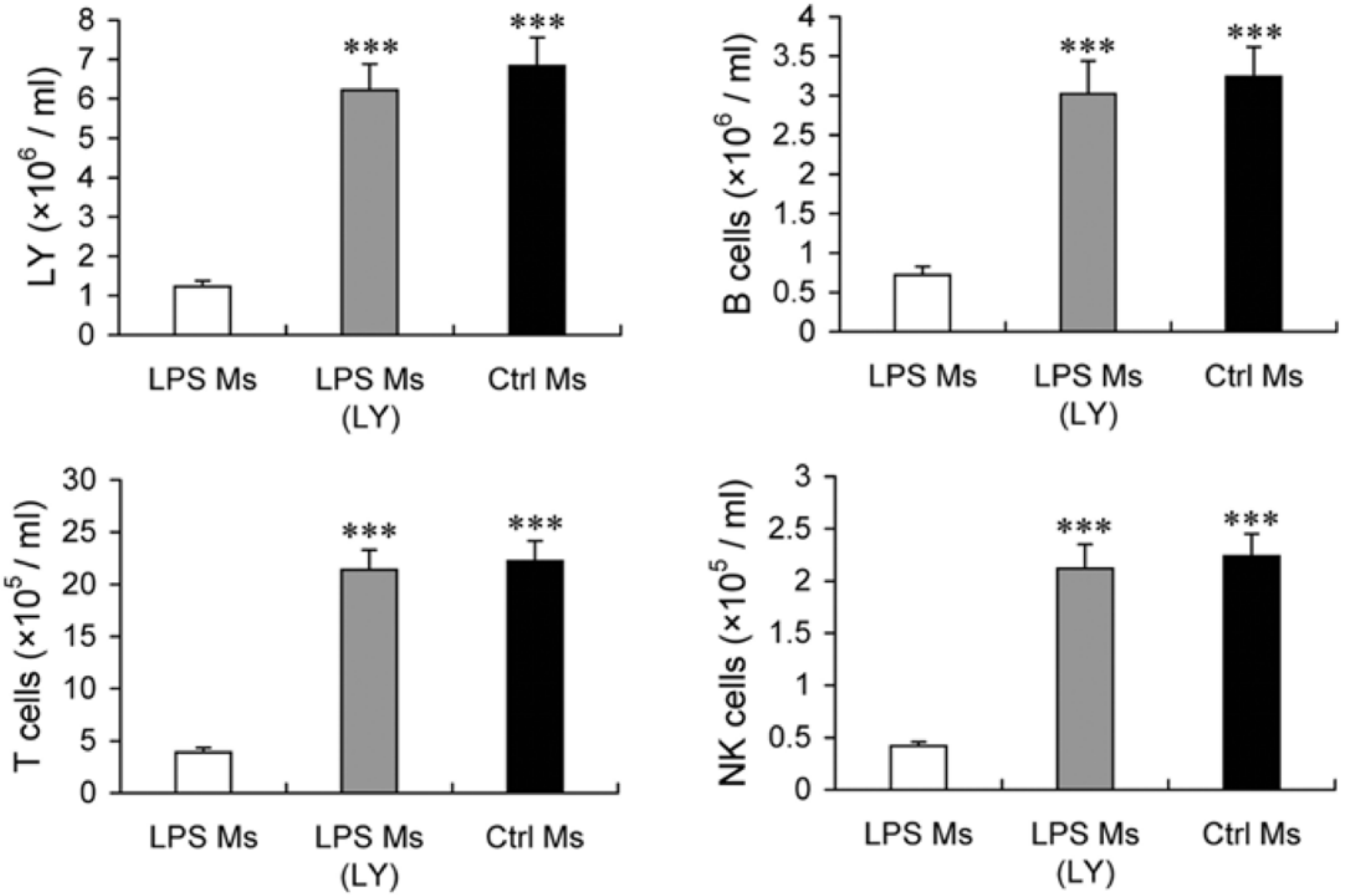

Supplementary Figure 3: Reconstitution of LPS mice with lymphocyte restores the levels of lymphocyte. Lymphocytes (LY) were isolated from spleens of normal mice and injected *i.v.* into LPS mice at 6 h post-CLP. Lymphocytes in recipient mice were confirmed at 24 h post-CLP by flow cytometry on blood samples from tail vein. The absolute number of lymphocytes was counted as described in Methods. The absolute number of lymphocytes in recipient LPS mice were not significant different from that in control mice. The results are means ± SEM values of 3 independent experiments (n=9). Data were analyzed by 1-way ANOVA with Bonferroni's multiple comparisons test; $^{***}p < 0.001$ versus the data in LPS mice.
